# The involvement of young people in school- and community-based noncommunicable disease prevention interventions: a scoping review of designs and outcomes

**DOI:** 10.1186/s12889-016-3779-1

**Published:** 2016-10-26

**Authors:** Didier Jourdan, Julie Hellesøe Christensen, Emily Darlington, Ane Høstgaard Bonde, Paul Bloch, Bjarne Bruun Jensen, Peter Bentsen

**Affiliations:** 1 Health Promotion Research, Steno Diabetes Center, Niels Steensens Vej 6, Gentofte, 2820 Denmark; 2 Laboratoire Acté EA 4281, ESPE Clermont-Auvergne, Université Blaise Pascal, 36, avenue Jean Jaurès C.S. 20001, Chamalières Cedex, 63407 France

**Keywords:** Community, Effect study, Health, NCD, Participation, Prevention, Pupil, School, Scoping study, Student

## Abstract

**Background:**

Since stakeholders’ active engagement is essential for public health strategies to be effective, this review is focused on intervention designs and outcomes of school- and community-based noncommunicable disease (NCD) prevention interventions involving children and young people.

**Methods:**

The review process was based on the principles of scoping reviews. A systematic search was conducted in eight major databases in October 2015. Empirical studies published in English, French, Portuguese, and Spanish were considered. Five selection criteria were applied. Included in the review were (1) empirical studies describing (2) a health intervention focused on diet and/or physical activity, (3) based on children’s and young people’s involvement that included (4) a relationship between school and local community while (5) providing explicit information about the outcomes of the intervention. The search provided 3995 hits, of which 3253 were screened by title and abstract, leading to the full-text screening of 24 papers. Ultimately, 12 papers were included in the review. The included papers were analysed independently by at least two reviewers.

**Results:**

Few relevant papers were identified because interventions are often either based on children’s involvement or are multi-setting, but rarely both. Children were involved through participation in needs assessments, health committees and advocacy. School-community collaboration ranged from shared activities, to joint interventions with common goals and activities. Most often, collaboration was school-initiated. Most papers provided a limited description of the outcomes. Positive effects were identified at the organisational level (policy, action plans, and healthy environments), in adult stakeholders (empowerment, healthy eating) and in children (knowledge, social norms, critical thinking, and health behaviour). Limitations related to the search and analytical methods are discussed.

**Conclusion:**

There are very few published studies on the effectiveness of interventions based on children’s involvement in school- and community-based NCD prevention programmes. However, interventions with these characteristics show potential benefits, and the merits of complex multi-setting approaches should be further explored through intervention-based studies assessing their effectiveness and identifying which components contribute to the observed outcomes.

**Electronic supplementary material:**

The online version of this article (doi:10.1186/s12889-016-3779-1) contains supplementary material, which is available to authorized users.

## Background

Noncommunicable diseases (NCDs)—mainly diabetes, cardiovascular diseases, cancer, and chronic respiratory diseases—are a large and growing public health challenge in high-resource and, increasingly, low-resource countries. It is therefore a critical issue to improve the effectiveness of prevention interventions aiming to enhance the health of children and young people and to reduce health inequities [1, 2].

Achieving intervention effectiveness requires an appropriate model of intervention as well as successful implementation, which should involve multiple and interlocked levels and components. Standard interventions, that often focus exclusively on modifying children and young people’s behaviour, are based on the implicit assumption that the locus of responsibility for childhood health problems lies with the children: if children are given the relevant information, they will adopt healthy, or healthier, behaviours [3]. There are two main limitations to this assumption. Firstly, most standard interventions consist of ready-to-use intervention programmes based on predefined activities, despite the consensus that the active involvement of a target group is crucial to the effectiveness of interventions [4]. Secondly, the health and well-being of children and young people are influenced by a complex interplay of biological, environmental, cultural, and social factors [5, 6].

First, with regard to active involvement of the target group, research shows that children’s high level of involvement in decisions about the design and implementation of interventions is critical for their effectiveness. It is also the case to improve health and health-related competencies, as well as for the sustainability of such programmes [4, 7]. Griebler et al.’s systematic review [7] identified evidence of the positive effects of student participation, especially in the students themselves. These effects included increased satisfaction, motivation and ownership, enhancement of skills, competencies and knowledge, personal development, health-related effects, and influence on student perspectives. In addition, positive changes at the organisational level of the school and in the interactions and social relations in the school setting were identified. These findings underscore the importance of active involvement of the target group in public health interventions.

Second, regarding social-environmental factors, literature shows prevention interventions should take into account the entire “life ecosystem” of children and young people. As a central component of this “life ecosystem”, school is an important setting for NCD prevention because children from all socio-economic and cultural backgrounds spend a large proportion of their waking hours there [8]. Also, schools as focal points in communities thus offer great potential in targeting risk factors related to behavioural, social and environmental health determinants on a wide scale [9–11]. However the link between schools and other settings and contexts is strong [12] and should not be discarded.

The authors of a Cochrane review called “Interventions for preventing obesity in children” [13] considered the following promising leads for effective interventions in schools: interventions targeting school curricula, physical activity sessions throughout the school week and healthy food supply in schools. In addition, environments and cultural practices should support and encourage children and young people to eat healthier foods and also support them in being active throughout each and every day. Capacity-building activities for teachers and other staff, and parental involvement were also important. A systematic review of studies on the effectiveness of school health promotion efforts, further concluded that programmes that account for contextual factors and emphasize multidimensional approaches are more likely to be effective in terms of health outcomes [14].

The increase in knowledge of health determinants has led to interventions becoming more complex [15], multilevel [16], and setting-based [17], striving for efficiency and transferability [18]. From the perspective of effectiveness, the key issue is to mobilise the diverse and valuable resources embedded in local community settings and to draw on the strengths of social interactions and local ownership as drivers of change processes. This strategy is embedded in the “supersetting” approach to mobilise local communities for public health action through coordinated and integrated engagement of multiple stakeholders in multiple community settings [12]. Initiatives in Denmark and Norway based on the supersetting approach have demonstrated important structural and behavioural outcomes, which are currently in preparation for publication.

Altogether, these findings from different multidisciplinary research fields suggest that a multi-setting and community-wide strategy based on stakeholders’ active involvement can be a relevant way to influence behavioural risk factors and prevent NCDs. The key idea is that children and young people can be agents of healthy change for themselves, their families and their communities [7].

Therefore, this scoping review had two aims. The first was to investigate the designs of multi-setting interventions involving children and young people as agents of change in schools and communities; the second was to examine the outcomes of these interventions to understand whether, and how, these interventions could lead to sustainable and effective NCD prevention in schools, families and local communities. This scoping review provides a literature map to identify the existing knowledge and gaps, and offers a summary of the state of the art and further research opportunities. Among the broad spectrum of interventions relating to NCD prevention, this work is limited to those targeting diet and/or physical activity.

## Methods

This scoping review [19] maps existing research literature that explores children’s participation as a component of interventions involving both schools and communities to improve health. The search was conducted in accordance with theoretical boundaries and working definitions identified from available literature reviews [7, 8, 11, 13, 14, 20–22] as well as previous work [3, 12, 23–26] as explained below.

### Working definitions

#### Intervention

In this review, we refer to an intervention as a “programme, service, policy or product that is intended to ultimately influence or change people’s social, environmental, and organisational conditions as well as their choices, attitudes, beliefs, and behaviour” [27] (p.452-453).

#### Prevention interventions in schools

Drawing from the Schools for Health in Europe network framework [28, 29], the term “schools” refers to all types of schools including nursery, primary and secondary, comprehensive, vocational, and specialised schools. Prevention interventions in schools could include all or some of the six components of a whole-school approach: healthy school policies, school physical environment, school social environment, individual health skills and action competencies, community connections, and/or health services.

#### Collaboration between school and local community

“Community” refers to the local community in which the school is anchored. Depending on the context it could be a village, district, or municipality; in other words, any territorial unit in which people have a collective sense of belonging and a shared identity [30, 31]. Collaboration between a school and the community entails elaborating and implementing a project relating to the early prevention of NCDs. The community includes a broad range of stakeholders, including parents, citizens, professionals and all types of public, private and civic organisations. The relationship between the school and the community may differ from one intervention to another, depending on the type of intervention [32].

#### Stakeholders

“Stakeholders” refers to all the people involved in the process of intervention implementation. Children are the most important stakeholders because they are the focus, and comprise the prime target group, of interventions. Professionals from the education, health and social sectors as well as parents, policy-makers and other adults in the school and local community are also stakeholders and often comprise secondary target groups.

#### Active involvement of stakeholders

The involvement of stakeholders in the intervention, also called participation in the intervention, implies that everyone with a stake in the intervention has a voice and an active role in the development and/or implementation process, with more or less influence on decision-making. People (e.g., children, parents, professionals, volunteers, and politicians) are considered to have the needed skills to act in the process. The level of involvement was defined with reference to Mygind, Hällman and Bentsen [26], Carmel, Whitaker and George [33] and Hart [34] regarding the “Ladder of Children’s Participation”. Involvement ranging from representative to consensus levels was considered (see the *Data analysis* section for details).

#### Outcomes

When considering complex interventions, based on a holistic view of health, that involve multiple stakeholders at various stages in the process of development and implementation, it is imperative to define what will be explored in terms of outcomes, i.e., what nature of outcomes is considered and how they will be evaluated. With regard to outcomes, we incorporated a wide range of effects, including health-related effects at the individual level of the participating children, the effects on adult stakeholders, and organisational changes and effects on the school and/or community setting.

### A scoping review

A scoping review can provide a relatively quick mapping of an area that is complex or that has not yet been subject to a comprehensive review. It maps the main sources and types of available evidence, as well as key concepts of emerging research areas [35]. This approach is relevant because the context studied is that of emerging evidence. “The difficulty of conducting systematic reviews of public health interventions directly reflects the complexity of the interventions reviewed and the subsequent determination of effectiveness. Some of the key challenges in the public health field include the focus on populations rather than individuals, multi-component interventions, qualitative as well as quantitative approaches, an emphasis on processes of implementation, and the complexity and long-term nature of the interventions and outcomes” [36] (p.367–368). A range of study designs were included in this review to address questions beyond those relating to intervention effectiveness, and to generate findings that can complement those of clinical trials [37].

### Search approach

We collected information about research on interventions addressing different levels of involvement and the development of children’s and young people’s capacity to critically explore and improve their physical environments, as well as initiatives targeting social factors influencing physical activity and healthy eating at different levels, e.g., the family, school, community, and city levels [3]. These interventions are based on a setting approach (i.e., school and community), and are designed to mobilise students and other local stakeholders involved in young people’s education and everyday lives.

### Database search

**Fig. 1:**
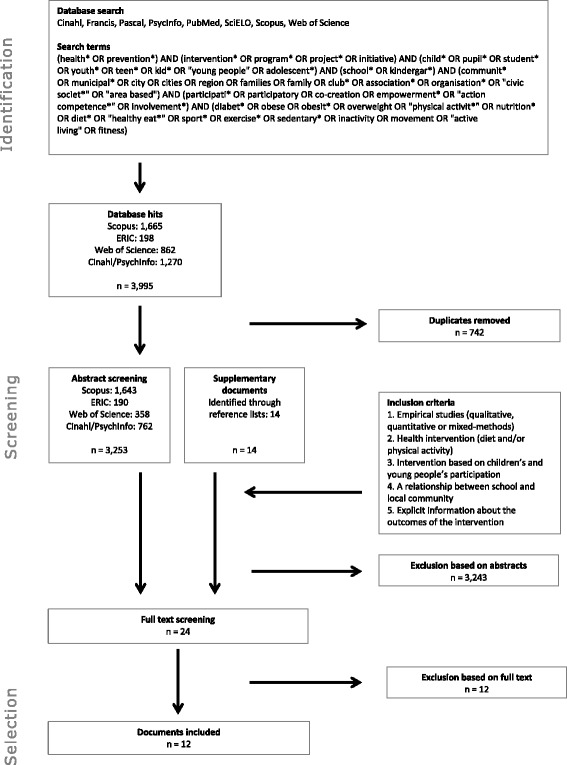
Search flow chart

The searches were conducted in October 2015. To identify relevant research-based evidence, a combination of several literature search strategies was used: electronic database queries, hand searching of key journals and consultation with experts and stakeholders. In addition, the reference lists of pertinent articles were checked for studies of relevance to the review. We reviewed papers in English, French, Portuguese, and Spanish. The focus was on empirical studies addressing children and young people’s involvement in health initiatives focused on physical activity and diet involving schools and communities. See Fig. 1 for the information sources and search terms.

### Inclusion criteria

Five selection criteria were applied. Included in the review were (1) empirical studies describing (2) a health intervention focused on diet and/or physical activity, (3) based on children’s and young people’s involvement that included (4) a relationship between school and local community, while (5) providing explicit information about the outcomes of the intervention on a structural, organisational and/or individual level. Papers in which one or more criteria were missing were not included.

### Screening process

A three-step selection process was applied. All search results (*n* = 3253) were included in a database, from which the first selection process was conducted by two researchers to determine the paper’s relevance for the review as assessed by title and abstract. Studies were excluded if the reviewers agreed that they did not meet the eligibility criteria (i.e., were not within the scope or contained insufficient information). Disagreements were resolved through discussion to reach consensus. Second, the selected articles (*n* = 24) were reviewed and assessed based on a full-text reading by two independent groups of two reviewers each, using a shared template to ensure internal validity. Papers for which there were no explicit references to participatory approaches and a school-community relationship were excluded. Third, the remaining papers were analysed by the two groups of researchers. The four reviewers then established the final sample, consisting of 12 papers.

### Stakeholder consultation

Three external experts (professionals from the health and education sectors) from Denmark and France were given the opportunity to suggest additional references, to provide insights beyond those found in the literature and to lend a critical view on the relevance of the findings.

### Data analysis

Descriptive information related to the study design and intervention design is available in the Additional file 1: Table S1. The analysis focuses on three types of intervention characteristics. Two of them relate to intervention design, namely the degree of children’s involvement and the nature of the school-community collaboration, while the third relates to intervention outcomes. The analytical approaches are described below for each analytical category.

#### Children’s involvement

Although there is a continuum of distinct definitions regarding children’s and young people’s influence and engagement, their levels of involvement were defined and applied in a deductive analysis. The categories in this review are inspired by Carmel, Whitaker and George’s [33] user-involvement spectrum—namely consultative, representative and consensus—and Hart’s [34] “Ladder of Children’s Participation”.

In the original work, there are three levels of participation. Only two were considered in this review, however, because the first (consultative) is characterised by children and young people solely providing and receiving information in relation to the intervention design, planning, implementation and/or evaluation processes. We do not consider this a genuine involvement of children and young people, since they are assigned and merely informed, while the school and/or community initiate and run the intervention.

The *representative* participation level is characterised by children and young people being consulted and informed, and providing suggestions to adult stakeholders regarding the initiative. They understand the process of implementation, their opinions are taken seriously, and they are informed as to how they have contributed to the final outcome. The suggestions provided by children serve to influence the adults’ decision-making; the children themselves do not directly act as decision-makers.

The participatory *consensus* level is characterised by children and young people taking responsibility for and influencing decisions relating to actions. Although the school and/or community may initiate the project, the decisions are shared with children and young people, whose wants and needs are articulated through an encouragement of their active involvement in the development process. Children and young people have real power and take part in designing, planning, implementing and/or evaluating the intervention.

#### School-community collaboration

The school-community relationship is categorised by three inductively defined levels of collaboration: (1) *Shared activities*, which refer to interventions with some degree of collaboration between schools and communities. Activities related to the intervention are present in both settings but without actual collaboration; (2) *Collaboration*, which refers to interventions with a high degree of collaboration in which either the school or the community takes the lead in developing and implementing the project. Actions may include partnership-building or communal initiatives; (3) *Joint intervention*, which refers to interventions based on a co-construction of the project throughout the process from design to implementation.

#### Intervention outcomes

Complex interventions may result in a wide range of organisational outcomes and effects on stakeholders involved in the project. The included studies were categorised in relation to the reported effects on one or more aspects of three stakeholder groups: (1) Structural outcomes at the community/school level. These include institutional changes in the community/school organisation; (2) Outcomes related to adult stakeholders, e.g., empowerment of stakeholders; Finally, the third category includes (3) Outcomes directly related to children, e.g., health-related effects, knowledge, and motivation.

## Results

### Overview of included studies

A total of 4009 publications were identified, and of these 3267 were screened after the removal of 742 duplicates. Based on the study participants and context, the interventions were designed and implemented according to a variety of reference frameworks: school health promotion, community health/healthy cities, and public health interventions addressing diabetes, obesity, physical activity, diet and eating habits and other related health issues. Of the 3267 screened papers, only 24 were selected for a full-text reading and in-depth analysis, 12 of which met our selection criteria and were ultimately included (Fig. 1).

The 12 included studies described 11 different interventions, two of which involved strong partnership-building between the school and community and a high level of involvement of children. Most of the interventions (eight) were mainly school-based but involved the community in various ways, whereas the remaining three were mainly community-based but involved schools (Table 1).

**Table 1:** Intervention design: involvement of children and school/community collaboration

Authors	Children and youth involvement		School/community collaboration		
	Representative	Consensus	Shared activities	Collaboration	Joint intervention
Birnbaum et al. (2002) [38]	Collecting children’s opinion: participation in health committees; School Nutrition Advisory Council (SNAC). Children are involved in the implementation of predefined interventions: peer leaders “help deliver the intervention”, i.e., a predefined curriculum.		Take-home activities and information: Parent Packs with a newsletter, tip sheets on healthy eating, and family assignments and activities.	Parents and staff take part in SNACs to develop policy practices.	
Carlsson & Simovska (2012) [47]		The intervention follows the Investigation-Vision-Action-Change (IVAC) approach and involves pupils in all its stages.			Local communities were involved in the health-promoting changes made in the school, e.g. through collaboration with community stakeholder consultants that take part in development and implementation.
Dzewaltowski et al. (2009) [40]	Children take part in school advocacy groups, “change teams”, led by adult site coordinators. Change teams create awareness of PA and healthy eating among peers. A curriculum supporting environmental change skills reached all involved classes. 14.9 % of intervention pupils take part in a change team. Children and youth take part in implementing change, but adults initiate and lead the intervention.		Shared activities between schools: school staff members receive training and are linked between schools in a “performance community hub” to facilitate sharing and problem-solving.		
Gådin et al. (2009) [44]		All pupils are involved in developing suggestions for school change in a participatory process using the “It’s your decision” model. A few pupils participate in an HPS committee and take part in the decision-making process. Students make up half the committee, which has the purpose of prioritising pupils’ proposals for environmental change and developing strategies for implementation.		Whole-school approach with involvement of parents in the school health committee. The amount of influence of the two parent representatives is unclear.	
Haapala et al. (2014) [41]	Involving children in implementation of predefined interventions: pupils take part in designing and executing recess activities as peer instructors and activators.				Schools and municipalities implement the plan that they have designed for themselves, backed by the national project framework.
Hannay et al. (2013) [49]		Teens are involved as co-researchers, taking part in defining framing questions, identifying problem areas and developing and implementing an action plan for advocacy for environmental and policy change in their community.		Presentation of the project by students to policy-makers and community stakeholders. Partnership between community members.	
Linton et al. (2014) [45]		Youth participating in the action groups perform community assessment and subsequently advocate for environmental and policy change with policy-makers. Genuine participation, including conducting surveys, assessing and prioritizing issues. Leadership and development and implementation of an action plan for advocacy for change in their community.		Adult mentors form youth action groups in schools and community centres. Mentors attend train-the-trainer seminars delivered by the programme.	
Orme et al. (2013) [39]	Participation in School Nutrition Action Groups (SNAGs); policy groups located at school. Pupil representatives collect the opinions of all pupils. Different levels of involvement in different schools. Pupils provide input through the SNAG, but decisions are made by school staff.			Whole-school approach including curriculum, school ethos and community involvement. Parents, teachers and school management participate in SNAGs.	
Ríos-Cortázar et al. (2014) [42]		Involving children in research: children as co-researchers. Children take part in leadership and advocacy: leadership and development and implementation of an action plan for advocacy for change in their community.		Partnerships: participatory approach involving students and the community. Three phases (exploratory, diagnostic, strategy definition).	
Rowe et al. (2010) [43]		A group of students conduct a survey among the other pupils, staff, parents and community stakeholders (the “ideal” school). Staff and senior students work together to address problem areas. Students take part in the visioning, advocacy, implementation and running of a “Kids Café”. Children contribute to Kids Café activities (recipes) and workshops.		Students, school staff, parents and community stakeholders take part in the launch of the initiative, a “Kids Café”, and the formation of a health committee. Parents and community stakeholders use the Kids Café and attend performances and educational activities. The area is established permanently through support from the community and local businesses.	
Simovska & Carlsson (2012) [48]		Involving children in decision-making and project management: participation as a learning-through-success strategy. Participation is used as an influence strategy that enhances confidence and competence.			Local communities are involved in the health-promoting changes made in the school. Changes to the local environment related to healthy eating in one school and PA in four schools. A “walking school bus” is established in collaboration with parents at two schools. Several schools collaborate with local authorities to improve local PA opportunities.
Toussaint et al. (2011) [46]		Youth Health Advocates (YHAs); high school youth enrolled in the programme receive training on health, leadership and more, to empower them to perform peer outreach. Youth are involved in implementation of activities. Power-sharing activities.			Community-generated programme including a community advisory board. The initiative includes a campaign and policy change efforts at local schools, and runs an after-school club.

Three studies were based on quantitative data collection methods, seven were qualitative, and two applied a mixed-methods approach. Most of them (eight) were case studies. Three papers describing multiple case studies were also included. Only one randomised control trial met the eligibility criteria and was included in the review.

### Children’s involvement

Concerning children’s involvement (Table 1), for four of the reviewed studies the participation level was categorised as *representative,* as children participated in health committees, a School Nutrition Advisory Council (SNAC) [38], School Nutrition Action Groups [39], or change teams [40]. This participation involved collecting children’s opinions and discussing problems and solutions as well as involving them in the decision-making process, but did not give them a lead role in the process. Some children were designated as “peer leaders” and were assigned to help deliver the curriculum [38] or activate other pupils [41] without assuming responsibility for the implementation. One study carried out surveys to capture children’s views [39] before they were involved in actual decision-making processes.

Eight studies described a *consensus*-level involvement of the children. In two of these, children were involved as co-researchers in addition to taking part in the entire implementation process [42]. The children themselves conducted a needs assessment and identified priority issues in three of the reviewed studies. For example, children conducted surveys and took part in suggesting changes to school issues relevant to their health [43, 44]; additionally, they defined problems to prioritise necessary and potential changes [44, 45], which enabled them to make decisions and develop health strategies themselves [44]. Children also implemented specific actions, for example a “Kids Café” and various workshops [43], and “power-sharing” activities [46]. Children’s involvement was described in three studies as leadership and advocacy, leading to the development of advocacy action plans [45] and an influence on strategies [47, 48] for change in the community [49]. Two studies applied explicit change models, namely the IVAC (Investigation—Vision—Action—Change) approach [48] and the “It’s your decision” model [44].

### School-community collaboration

Stakeholders from schools and local communities were involved in interventions in various ways, illustrating different levels of integration of the two settings. The characteristics of the school-community relationships are illustrated for each study in Table 1.

Two interventions were centred on *shared activities* between schools and communities. In one study, take-home activities, information and advice were mailed to parents several times during the project period to support the classroom curriculum [38]. Alternatively, shared activities between schools, e.g., through staff training sessions, were part of the creation of a community hub [40].

The second level, *collaboration*, included schools that invited the local community, including parents, to take part in activities on the school premises [43]. Six studies fell within this category. In some cases, this was conducted through participation in health committees and action groups [38, 39, 44]. In one study, parents and youth collaborated to identify barriers to physical activity in their school and local community [49].

Finally, *joint interventions* were identified for three interventions in which schools and communities identified and addressed health barriers together. For example, community stakeholders took part in the development and implementation of plans for action [47]. Two interventions in this category were school-initiated [41, 47, 48], whereas the third was initiated by the community [46].

### Intervention outcomes

Table 2 shows the three categories of outcomes identified. Ten of the 12 reviewed studies reported on outcomes related to the *children*. This included behavioural outcomes, e.g., an increase in physical activity [40, 41, 46] and healthy eating [38], as well as positive changes in social and personal skills, e.g., leadership and advocacy skills [43, 49], critical thinking [46, 47], and changes in attitudes [42] and social responsibility [48].

**Table 2:** Intervention outcomes for children, adult stakeholders and schools/communities

Authors	Outcomes for children	Outcomes for stakeholders	Outcomes for schools and communities
Birnbaum et al. (2002) [38]	Impact on fruit and vegetable consumption (from 4.88 + -0.06 servings to 5.80 + -0.05) and food choice (from a score of 5.90 + -0.16 to 6.54 + -0.16) for those exposed to environment changes, curriculum intervention and being peer leaders.		
Carlsson & Simovska (2012) [47]	Changes in pupils’ action competence through increased knowledge (related to healthy behaviours and health determinants), self-confidence, communication skills and critical thinking.		Changes in school meal provision and PA-promoting environments, e.g., bicycle parking lot, road safety on the school and community levels.
Dzewaltowski et al. (2009) [40]	Intervention schools increased in PA while controls decreased from years 7 to 8. 3.7 % increase in physical activity after school, corresponding to an increase of 7.5 min per day. FV intake did not change over time compared to controls.	Self-efficacy of adult leaders was high before the intervention and remained high at all measurements.	
Gådin et al. (2009) [44]	The outcomes reported are not the result of an evaluation, but rather the expected outcomes based on the changes made. These include empowerment and increased influence on their school life, leading to mental health benefits.		Change in school and community policy: adaptation of existing policies, rules and action plans, e.g., action plan against bullying. Physical changes to the school playground to increase PA and improve social relations between pupils.
Haapala et al. (2014) [41]	Increase in recess physically active play (from 30 to 49 %) and ball games (from 33 to 42 %) during the project, mainly due to males’ participation. However, PA decreased in the follow-up period. Pupils who spent recess outdoors increased from 17 to 33 % in the project period.		Change in the organisation of the school day, including more opportunities for PA. Development of facilities and equipment for PA during the project. At one school, networks with parents and municipality office-holders were established for PA promotion.
Hannay et al. (2013) [49]	Development of advocacy skills, enhanced self-esteem and confidence, and motivation to engage in further advocacy.	Parents reaped personal benefit from contributing to overcoming negative stereotypes.	Advocacy by participants led to changes in policies for credit towards physical education in an alternative setting and changes to a school bus route.
Linton et al. (2014) [45]			Implementation of environmental changes in schools and communities following advocacy activities e.g., extra lighting, salad bar, female-only swim time.
Orme et al. (2013) [39]			Improvements to school meals and dining environment reported by pupils.
Ríos-Cortázar et al. (2014) [42]	Changed behaviour, attitudes and norms: the programme had an impact on children’s cognitive, social and emotional levels, nutrition and physical activity.		
Rowe et al. (2010) [43]	The learning process developed pupils’ advocacy skills.		Stronger relationship between school and community. The intervention ensured the availability of healthy, affordable meals through the establishment of the Kids Café and supported an environment that promotes healthy eating behaviours.
Simovska & Carlsson (2012) [48]	Skills and competence: leadership increased the sense of responsibility and motivation in pupils, development of learning and competency. Development of social responsibility, e.g., considering younger peers.		Provision of healthy eating and PA opportunities and improved environment e.g., bicycle parking lot, road safety initiatives on the school and community levels.
Toussaint et al. (2011) [46]	Change in eating habits, e.g., less sugar intake, and increase in PA. Development of critical thinking, leadership and advocacy skills, enhancement of self-esteem and confidence, motivation to engage in higher education.	Change in family members: healthier eating habits, weight loss, increased PA.	

In regard to adult *stakeholders*, their mobilisation and involvement were not only intermediate secondary outcomes but crucial requirements for the implementation and effectiveness of complex prevention interventions. Only three studies offered some description of personal outcomes of stakeholders’ involvement in the interventions, including healthy changes in family members’ lifestyles [46], and personal benefit in relation to overcoming stereotypes [49].

Eight papers reported *outcomes for schools and communities*, most of which entailed changes in the school setting. For example, Gådin, Weiner and Ahlgren [44] reported on the adaptation of existing school policies, rules and action plans, e.g., an action plan against bullying. Haapala et al. [41] reported on changes in the organisation of the school day, including providing more opportunities to be physically active, and Linton and colleagues [45] observed that children engaged in advocacy with decision-makers, leading to concrete structural changes, e.g., extra lighting, the establishment of a salad bar and female-only swim-time. Other studies found changes in the school and community environment, e.g., building a bicycle parking lot and taking road safety measures [47, 48].

### Impact of involvement of stakeholders on the outcomes of the intervention

In some studies, empowering children and increasing their leadership and advocacy skills for health were the main desired outcomes of the intervention [46, 47]. In these cases, the active involvement of children and youth not only affected the intervention; it was crucial to achieving its purpose. In a school setting, real-life experiential learning activities [43] led to outcomes related to self-efficacy and leadership skills which support healthy behaviours and are health enhancing in themselves. In their dose-response study, Birnbaum et al. [38] showed that positive outcomes related to healthy eating were only achieved for the group of pupils who were involved the most (as peer leaders).

Adult stakeholder involvement often consisted of parent participation in a health committee [39], meaning that only very few parents took an active part in the intervention. Their actual influence on the implemented activities remains unclear. Further, the outcomes for community stakeholders are rarely explored, including effects on parents of being the recipient of health messages delivered through their children. The changes made to the local environment, based on children’s participation, may potentially influence the lifestyles of other community members; however, this question is not explored in the included studies.

A majority of the studies reported changes to the school and/or community setting, such as policy development and physical changes. These outcomes are the result of the participatory processes involving both children and adult stakeholders. The power relations in play and the extent to which children had a genuine influence on the entire process differ between studies and are not necessarily explicit. Based on the included studies, we cannot make conclusions as to the effect of the organisational changes, or the relationship between different levels of children’s involvement and the observed health outcomes.

## Discussion

### Results and limitations of the included studies

In this scoping review of the designs and outcomes of school- and community-based prevention interventions involving children and young people, positive effects were observed in some interventions. These effects were related to organisational change (e.g., adaptation of existing policy, rules and action plans, changes in school and community environments, and relationships between schools and communities), stakeholders (e.g., empowerment, healthier eating habits) and children (e.g., knowledge, views on social norms, critical thinking and healthy behaviour regarding the consumption of fruits and vegetables).

Although all studies, in accordance with the inclusion criteria, involved both schools and the community, it is worth noting that schools initiated most of the initiatives and, additionally, that community involvement in several studies consisted primarily of collaboration with parents rather than the wider community. Interventions founded on collaboration and partnerships between schools and community stakeholders such as local organisations, non-governmental organisations, and companies are thus scarce, as are community-based interventions working in and with schools.

The review included and analysed very few studies, most of which barely met the inclusion criteria; this is not surprising, however, considering our rather specific criteria. The overall quality of evidence must therefore be regarded as low to moderate. Moreover, the included studies had certain methodological limitations, including their reliance on self-reported data and the lack of long-term follow-up data for most of them.

The small number of studies meeting our five rather specific inclusion criteria is due to two main reasons. The first is linked to the fact that most of the excluded papers did not describe interventions including a multi-setting approach, either school-based or community-based; and when they did involve both the school and community settings, the approach was rarely participatory. For example, on the one hand, the seminal reference work called EPODE, which aimed to mobilise stakeholders on all levels across public and private sectors [50], did not rely on the active involvement of children and young people. The same held true for the highly influential, multifaceted community capacity-building interventions Shape Up Somerville [51] and Be Active Eat Well [52]. On the other hand, interventions based on children’s involvement, such as those implemented in Avall 2 [53], did adopt a whole-school approach but did not report on a school-community partnership.

The second reason is that when the interventions did meet the inclusion criteria, the description of outcomes was often weak or non-existent [54], and the paper was therefore excluded from our review, as explained in the section on the selection processes. We note that the apparent lack of such characteristics may be a result of the reporting process rather than the study itself, e.g. publishing several papers from one intervention with different focusses, e.g. the design vs. the outcomes of the intervention and the school vs. the community part of an intervention.

### Implications for practice, policy and research

The present review highlights a research gap related to the (rather limited) number of studies applying a combination of a participatory approach involving children and a school- and community-based intervention, while also describing the outcomes of the intervention. In addition, participatory interventions conducted within the framework of combined school-community partnerships are scarce, and this scarcity suggests that the research community involved in designing interventions and evaluating their effects, as well as policy makers and practitioners implementing interventions, have still not capitalised on the available opportunities and resources within different fields. It appears as though there are barriers to the creation and implementation of interventions based on state-of-the-art knowledge from the education sector (e.g. pedagogy and whole-school approaches), the public health sector (e.g. community-based approaches) and the field of theory-based evaluation of complex and multilevel interventions (e.g. rigorous evaluations of outcomes). We thus identified gaps related to the combination of four characteristics of prevention interventions targeting children and young people:

- A community-based approach to mobilise the diverse and valuable resources available in local community settings, and to draw on the strengths of social interactions and local ownership as drivers of change processes [12].
- A whole-school approach [55] that includes coherence between school policy and practice to improve social inclusion and commitment to education, to facilitate the improvement of learning outcomes and to increase emotional well-being and reduce health risk behaviours [11]. This approach is most effective when the school uses its full organisational potential to enhance health among students, staff, families and community members [56].
- A participatory approach addressing health and actively involving children as well as various other groups of stakeholders in the processes of influencing decision-making regarding the design, planning, implementation and/or evaluation of interventions [57]. This approach means that the emphasis is not on implementing interventions “targeting children” but rather on creating the necessary conditions to reduce risk factors and enhance children’s health. The mobilisation, active involvement and empowerment of stakeholders are not only intermediate outcomes but also critical preconditions for the effectiveness of prevention efforts and are therefore goals in and of themselves [58, 59].
- A relevant evaluation framework that has proven useful in assessing the quality of implementation processes while determining the effects of interventions. Many frameworks and approaches have attempted to identify the components of effects of complex interventions (e.g. the RE-AIM framework [60], cost-effectiveness models, and multiplier assessments [61] and the most relevant level to evaluate them (e.g., individual, group, setting, and/or institutional level [62]. To provide a dynamic account of the inherent complexity of interventions, theory-based realistic evaluations have been identified as a relevant framework of choice to evaluate interventions implemented in complex school- and/or community-based settings.
- In addition to these gaps, it would be interesting and highly relevant to investigate and compare the effectiveness of “outcomes” between studies that involve children and young people in interventions and those that do not. Related to this, future research should explore methodological issues related to how one measures the effectiveness of the active involvement of children and stakeholders.

### Strengths and limitations of this review

The systematic scoping process, including a thorough search, the inclusion of papers in languages in addition to English, and the consultation of expert stakeholders, is a methodological strength of this review. However, the methods applied also entail some limitations. As the level of analysis in the review was papers rather than interventions, relevant interventions may have been missed in the initial search, if the design and outcomes of an intervention were described in separate papers. Furthermore, our analytical categorisations were based on information available in the included papers and may thus represent a simplification of the actual intervention processes. Despite these limitations, we consider the review a relevant contribution to this complex field.

## Conclusions

In conclusion, this scoping review highlights that there are very few published studies on the effectiveness of interventions based on children’s and young people’s active involvement in combined school- and community-based NCD prevention programmes. Nevertheless, it shows the potential benefits of complex and multilevel interventions implemented within the framework of community- and setting-based strategies that incorporate a strong and well-defined participatory approach. Due to the weakness of the available evidence, further intervention-based studies [63] should be developed and implemented to properly assess the effectiveness of such interventions and to identify which components contributed most to the effects observed. To be conclusive, such projects should be built on a strong theoretical basis and must be implemented in genuine partnership between researchers and professional stakeholders from the education and health sectors, e.g. practitioners and policy-makers.

## Additional file

Additional file 1: Table S1. Overview of studies. (DOCX 29 kb)

## Abbreviations

NCD: Noncommunicable disease
PA: Physical activity
