# Supplementary material for: The involvement of young people in school- and community-based noncommunicable disease prevention interventions: a scoping review of designs and outcomes
Source: BMC Public Health. 2016 Oct 26;16:1123. doi: 10.1186/s12889-016-3779-1 (PMC5080716; doi:10.1186/s12889-016-3779-1)
Supplement: Additional file 1: Table S1. — Overview of studies. (DOCX 29 kb) [file 12889_2016_3779_MOESM1_ESM.docx]

**Table S1. Overview of studies.**

| **Authors and country** | **Study type, design and methods** | **Participants and setting** | **Aims and purpose** | **Intervention** |
| --- | --- | --- | --- | --- |
| **Birnbaum et al. (2002)**  USA | School-based group-randomised trial: 16 schools: 8 intervention, 8 control. 2-year implementation period. Dose response with four levels of intervention: (1) control group (2) school environment interventions (SE) only, (3) classroom curriculum (CC) plus SE, and (4) peer leaders plus CC plus SE (highest exposure). Student surveys (*n*=3,503 for baseline and follow-up) assessed eating habits and psychosocial mediators, barriers, social norms and intentions related to healthy eating. | One pilot school: 16 middle and junior high schools participated in the intervention. Baseline in 7^th^ grade (3,878 students). End-line in 8^th^ grade (3,503 students). | To evaluate school-environment, classroom, and family interventions to increase fruit and vegetable (FV) intake and decrease fat intake of low-income youth to reduce their future risk of cancer. To examine outcomes on students’ eating patterns and their association with different levels of exposure. | A school-based nutrition intervention (“TEENS”) with different levels of intervention exposure within each school. Peer leaders helped deliver the intervention by leading small-group activities and discussions. Ten curriculum sessions on healthy eating behaviour. Self-assessment and goal setting with progress checks. Students receiving the curriculum also received three “Parent Packs” with activities and intervention-related messages. The school environment intervention promoted FV as part of the school lunch and FV and lower fat foods as healthy snacks available at schools. |
| **Carlsson & Simovska (2012)**  Austria, Denmark, Italy, the Netherlands, and Spain | Qualitative multiple case study with cross-case analysis. Data: (1) project documents (2) content on the project’s web portal (3) observations made on two school visits (2–3 days per case), (4) interviews with local coordinators (*n* = 10), (5) interviews with teachers (*n* = 10), and group interviews with six students in each school (*n* = 30). Learning outcomes/action competence assessed through teacher interviews. | Lower secondary schools (ages 11-16) were selected from a total of 73 Shape Up schools in 19 EU countries and cities. Five schools in different countries participated: two small schools (200 students), two medium-sized schools (500–600 students), and one large school (1,100 students). | To develop children’s capacity to critically explore and implement healthy changes at school and in the community. To explore learning outcomes, i.e., changes in children’s action competence in relation to health and mechanisms conducive to these changes. | Shape Up: a school-community approach to influencing determinants of healthy growing up. Applies the IVAC approach (Investigation, Vision, Action, and Change) and focuses on healthy eating and physical activity (PA). School projects include activities decided on by students: e.g., establishing a playground and changing school food options and activities for students planned by adults. Development and implementation by local partners in collaboration with the international project partners. |
| **Dzewaltowski et al. (2009)**  USA | Randomised control trial. Nested cohort design with a priori stratification and school as the unit of randomisation. Schools were assigned to three school strata for randomisation by students’ socio-economic status, ethnic diversity and school size. Measures at baseline, year 1 and year 2: youth self-report of PA, FV, BMI, and psychosocial variables with validated questionnaires. Implementation evaluation by self-report: survey assessed coordinators’ self-efficacy to lead and train others and they reported on change team meetings by logging implemented programmes, policies, and environmental changes. Teachers reported on curriculum implementation. Youth survey of awareness of and participation in project activities. | Eight intervention schools (815 students) and eight control schools (767 students), in total 1,582 participating middle school students in grades 6-8. | To evaluate the effectiveness of a multilevel intervention model designed to develop the skills and efficacy of adult leaders and youth to create middle school environments (healthy places) that promote FV and PA. | Multilevel intervention model to build skills and efficacy of youth and adult leaders to create PA- and FV-promoting school environments. Group staff training of site coordinators. Network for coordinators to facilitate sharing and problem solving. School change teams (youth and adults) to create awareness of health messages in school. Coordinators facilitated participatory planning by targeting a place for environmental change, developing objectives that appealed to youth, and focusing on building youth’s skills or providing options for PA or FV in a positive social environment. Curriculum for 7^th^ and 8^th^ grade students on the planning process and youth social, communication and environmental change skills to help facilitate student leadership. |
| **Gådin et al. (2009)**  Sweden | Case study of an intervention based on a participatory action research approach. Analysis of students’ group discussions about health priorities and follow-up discussion in a health committee. Analysis of changes planned by health committee. Data: 41 proposals for change from students, developed during group work, and documentation from eight health committee meetings. | One elementary school with grades 1-6 students (app. 150). The school is located in a relatively low-income area. | To explore whether young students could be substantive participants in a health-promoting school (HPS) project. To analyse changes to school environments proposed by students and how changes were prioritised by a school health committee. | Five-day introduction for teachers with information about health, gender theories and gender pedagogy and the health educational model, “It’s your decision”. Thematic weeks to increase gender awareness among students and meetings with small-group discussions about health promoting (HP) factors: students’ role as classmates and qualities as a school friend. Students proposed health-enhancing school changes. A health committee with students, parents and school staff members prioritised students’ proposals and developed strategies to improve the school environment. |
| **Haapala et al. (2014)**  Finland | Quantitative study. Anonymous self-report survey of PA and demographic variables was conducted four times in the same classes. Local contact persons reported school activities, theme days, meetings and active commuting to school four times during the follow-up period. Telephone interviews and internet surveys with seven local contact persons to account for their perceptions of changes were analysed quantitatively. | Four lower secondary schools. Data collected from students in grades 7–8 and the following year in grades 8–9. Schools varied in size and location. Response rates at the four measurements: 704-791 students (45-47% male). | To promote school-based PA. Study aims: (1) to assess changes in adolescents’ recess and overall PA. (2) To describe the promotion actions in four schools participating in the programme. (3) To explore which effects local contact persons perceived of these actions on students’ PA. | Four schools participating in the national Finnish Schools on the Move action programme were studied. The programme uses a bottom-up approach by encouraging participating schools and municipalities to implement their own specific plans to increase PA during the school day. At national programme seminars, schools were provided with further ideas for activating school days. Schools could cooperate with an experienced mentor to support and help them in the promotion process. |
| **Hannay et al. (2013)**  USA | Qualitative case study. Midcourse evaluation of an afterschool obesity prevention programme. Nine focus groups were conducted, four with adolescent girls (*n*=36) and fıve with parents (*n*=41). These were supplemented by Photovoice workshops: three with teens and two with parent–teen dyads. | The study is based in a community health centre. 25 Latino participants: 19 teens, six adults. | To identify Latina teens and their parents’ barriers to PA and initiate policy change actions to address them through participation. To assess Photovoice as both an evaluation tool and an advocacy intervention in a community-based obesity prevention programme. | An after school obesity prevention programme, Healthy Tomorrows for New Britain Teens. The intervention combined Photovoice (a community-based participatory research methodology) with focus groups to engage Latina teens and their parents in identifying barriers to physical activity and initiating policy change actions. The effectiveness of applying Photovoice as both an evaluation tool and a leadership/advocacy intervention is assessed. |
| **Linton et al. (2014)**  USA | Case study using mixed methods. (1) Interviews with decision-makers to assess perceptions of interactions with teenagers. (2) Adult leaders’ survey to assess group characteristics, needs, process information and factors to success. (3) Teenagers’ surveys (pre and post) to assess changes in attitudes, behaviour and perception of control, efficacy and sense of advocacy. Baseline surveys of youth demographics. | Twenty YEAH! groups participated; 6 in high schools, 6 in middle schools, 8 in community centres. Data were collected from youth participants (*n*=136), adult group leaders (*n*=18) and decision-makers. | Evaluation objectives: documenting group process and success of engaging in community advocacy with decision-makers and to note preliminary successes in securing policy and environmental change as a result of group efforts. | Youth Engagement and Action for Health! (YEAH!), a youth advocacy and community-empowerment programme, engages local youth and adult mentors in advocating for improvements in opportunities for PA and local access to healthy foods. YEAH! engages youth in assessing their environments, prioritising problems based on those assessments, and developing and implementing an action plan to advocate with decision makers for changes. |
| **Orme et al. (2013)**  England | Case study with a mixed methods approach. Surveys distributed to lead teacher contacts at the 75 schools with a follow-up questionnaire after 18-24 months. Interviews with lead staff (*n*=24). Interim data collection at six schools after one year with staff interviews (*n*=12) and focus groups with students (*n*=77) to elicit perceptions of implementation and involvement. | Seventy-five primary schools participated in the Food for Life Partnership programme (open to primary, secondary and special schools). Study participants were 77 students (year 3 and year 5) from six schools. | To examine students’ role in the implementation of a whole school food programme by exploring (1) the nature and extent of student participation; (2) perspectives of school staff; (3) perspectives of student participants; (4) implications for policy, practice and research. | The Food for Life Partnership programme is a multi-level initiative using a whole-school approach to promote healthier nutrition and food sustainability awareness for students and their families. Initiatives include involving children in cooking, growing, farm visits and School Nutrition Action Groups (SNAGs). Schools focus on improvements in quality of school food provision, health education, participation, collaboration and structural and policy change. |
| **Ríos-Cortázar et al. (2014)**  Mexico | Participatory action research, which was adapted and integrated into a participatory method of planning-action-evaluation, with a model of collaborative and group learning. | One primary school in southern Mexico. The background of the school was highly disadvantaged. | Nutrition, physical activity, sedentary lifestyle and other health issues | HPS initiative. Participatory approach involving students and community. Three phases (exploratory, diagnostic, strategy definition). Students’, parents and staff’s views were taken into account. Publication of a journal. |
| **Rowe et al. (2010)**  Australia | Qualitative case study. 23 interviews with students, teachers, parents and staff. Four focus groups with students (age 8-12). Observations and document analysis. Process evaluation.  Data were collected using in-depth interviews, student focus groups and documentary evidence, such as school planning documents and observations of HP school activities. | One small primary school. The school was selected from a broader study of schools implementing the HPS approach based on its commitment to HPS. Study participants: 23 school representatives and app. 25 students aged 8-12. | To foster improved nutrition in schools by creating a supportive environment for healthy eating. To investigate the influence of a HPS approach on nutrition improvements in the school environment. | Whole-school approach. 19 students aged 11-12 years conducted a survey representing the views of the 300 students, school staff members, parents and the broader community. The results were used to form a vision of the school community’s “ideal” school, focusing on healthy nutrition. A HPS committee identified priority areas and made action plans. A “Kids Café” was established to provide a healthy meal for sale for school members and the wider community in a social environment. |
| **Simovska & Carlsson (2012)**  Austria, Denmark, Italy, the Netherlands, and Spain | Qualitative cross-case study involving five cases to explore processes leading to change, participation levels and challenges. Data sources: documents (project reports, self-evaluations etc.) and web content. Observations made on two school visits (2–3 days per case). Interviews with coordinators (n=10). Group interviews with students age 12-16 (*n*=30) and individual interviews with teachers (*n*=10). | Shape Up intervention in 19 European cities (73 schools, 2300 students, 140 teachers).  Study participants: School staff (*n*=20) and students age 12-16 (*n*=30) from 5 schools (Austria, Denmark, Italy, the Netherlands, and Spain). | Shape Up aims to integrate participatory health education, disease prevention and HP in an intervention. Study aims: to discuss the outcomes of the Shape Up intervention and challenges to students’ participation in school HP. | Schools took part in the HPS project, Shape Up. The participatory IVAC approach (Investigation, Vision, Action, and Change) was applied to facilitate HP changes related to PA and healthy eating through participation, action-orientation, structural changes and collaboration. |
| **Toussaint et al. (2011)**  USA | Qualitative case study. Semi-structured interviews with teenagers (*n*=6) about their motivation to become a youth advocate and the impact of their participation as a youth advocate on themselves and their relatives. | Six students (age 14-18, four female) selected by the YHA (youth health advocate) Outreach Coordinator to participate. | To reduce the prevalence of diabetes among Latinos in the community. To explore why Latino high school students decide to become YHAs, impacts on their own and their family’s health, and the impact on leadership ability and self-confidence. | A community-generated initiative with a community advisory board. The initiative includes an active *Promotoras* campaign and policy change efforts at local schools. Additionally, a YHA afterschool club at community high schools was created, developed and supported. YHAs receive training on health, leadership, and digital storytelling, empowering them to undertake outreach to peers. |
